# Microstructure of the retinal pigment epithelium near-infrared autofluorescence in healthy young eyes and in patients with AMD

**DOI:** 10.1038/s41598-020-66581-x

**Published:** 2020-06-12

**Authors:** Kari V. Vienola, Min Zhang, Valerie C. Snyder, José-Alain Sahel, Kunal K. Dansingani, Ethan A. Rossi

**Affiliations:** 1 Department of Ophthalmology, University of Pittsburgh School of Medicine, Pittsburgh, PA 15213 USA; 2 Department of Bioengineering, University of Pittsburgh Swanson School of Engineering, Pittsburgh, PA 15213 USA

**Keywords:** Retinal diseases, Biomedical engineering, Optical techniques

## Abstract

Retinal pigmented epithelial (RPE) cells are essential for maintaining normal visual function, especially in their role in the visual cycle, and are thought to be one of the first cell classes affected by age-related macular degeneration (AMD). Clinical imaging systems routinely evaluate the structure of the RPE at the tissue level, but cellular level information may provide valuable RPE biomarkers of health, aging and disease. In this exploratory study, participants were imaged with 795 nm excitation in adaptive optics scanning laser ophthalmoscopy (AOSLO) to observe the microstructure of the near-infrared autofluorescence (AO-IRAF) from the RPE layer in healthy retinas and patients with AMD. The expected hexagonal mosaic of RPE cells was only sometimes seen in normal eyes, while AMD patients exhibited highly variable patterns of altered AO-IRAF. In some participants, AO-IRAF structure corresponding to cones was observed, as we have demonstrated previously. In some AMD patients, marked alterations in the pattern of AO-IRAF could be seen even in areas where the RPE appeared relatively normal in clinical imaging modalities, such as spectral domain optical coherence tomography (SD-OCT). AO-IRAF imaging using AOSLO offers promise for better detection and understanding of early RPE changes in the course of AMD, potentially before clinical signs appear.

## Introduction

The human retina naturally emits light as it is absorbing light due to its intrinsic autofluorescence (AF). Over the past three decades, several imaging modalities, such as the scanning laser ophthalmoscope (SLO), have been utilized to visualize the AF of the retina^1–4^. Clinically, fundus autofluorescence (FAF) has become routine for evaluating disease status and monitoring progression in certain pathologies because of its potential to reveal retinal pigment epithelium alterations that are difficult to distinguish using other imaging modalities^4–6^. It has also been capitalized on in adaptive optics scanning laser ophthalmoscopy (AOSLO) to image individual retinal pigmented epithelial (RPE) cells^7–9^. However, many questions remain about the origin of retinal AF and its usefulness for visualizing the RPE cell mosaic in aging and in diseases of the outer retina, such as age-related macular degeneration (AMD). Fundus AF originates from the retinal pigment epithelium and choroid. Though choroidal AF is thought to arise primarily from melanosomes and melanocytes, RPE cells contain additional AF organelles, including lipofuscin, and melanolipofuscin (complex aggregates of melanin and lipofuscin)^10^.

Fundus autofluorescence has been evaluated at several imaging wavelengths across the visible spectrum and into the near-infrared (NIR). When using short wavelength autofluorescence (SWAF) excitation (i.e. visible wavelengths shorter than 700 nm), the AF signal has been thought to originate primarily from lipofuscin. Lipofuscin is a complex aggregate of bisretinoids, some of which are autofluorescent, that accumulates in RPE cells over the lifetime^11,12^ because of the visual cycle^13,14^. SWAF has been shown to increase with age^15,16^ and in some retinal diseases^17,18^. With NIR excitation, melanin has been thought to be the main source of the AF signal^4^. However, ocular melanin and its emission spectra are not well researched, with most spectral data coming from non-ocular sources of melanin^19,20^. Over time, it is thought that lipofuscin increases^21,22^, and pure melanin decreases^23^ as they form complex aggregates called melanolipofuscin. Interestingly, new evidence by Taubitz *et al*. suggests that melanin degradation products may also be incorporated into lipofuscin with age as they showed that lipofuscin granules exhibit both SWAF and IRAF in aged human donor eyes^24^. This finding helps explain our previously published report showing co-localization of SWAF and IRAF signals in AOSLO^9^. However, it remains unknown how alterations in the abundance and composition of RPE fluorophores with aging and disease alters the AF spectra of RPE.

NIR excitation presents some potential advantages for clinical applications. It is inherently safer than short wavelength excitation, as it only presents thermal safety concerns, whereas both thermal and photochemical damage are of concern for shorter wavelengths^25^. NIR light is also more comfortable for patients to view, owing to the greatly reduced spectral sensitivity of the photoreceptors to this wavelength range. However, the signal strength of AO-IRAF is substantially lower than SWAF^4^ and it is unclear if the increased safety profile for AO-IRAF is enough to overcome this limitation. Though it has been suggested that AO-IRAF offers complementary information to SWAF^26^ and may be a promising alternative for studying early cellular alterations to the RPE in diseases such as AMD, to date it has only been visualized at a cellular level in a small number of eyes in a limited age range and in only a few diseased eyes^9,27,28^. Thus, it remains unknown whether AO-IRAF can effectively and routinely be used to image RPE cells in normal eyes or eyes with AMD. Understanding its strengths and limitations for visualizing RPE alterations in AMD is the primary objective of the present study.

AMD is a progressive disease of the outer retinal neurovascular unit consisting of the RPE, Bruch’s membrane and the choroid^29,30^. The earliest stage of the disease is asymptomatic and is usually diagnosed when extracellular aggregates called drusen are detected in sufficient size and number clinically. The transition from early to intermediate disease is defined by the formation of larger drusen and/or the emergence of pigmentary abnormalities. The latter are difficult to quantify and consist of funduscopically visible hyper- and/or hypo- pigmentation of the RPE. Late stage AMD can be neovascular, where new vessels grow from the choroid towards the outer retina. They leak fluids below the retina that can cause photoreceptor death and lead to severe vision loss^31^. However, the vast majority of cases of AMD (~90%) remain non-neovascular^32^, for which no treatment exists. Late stage non-neovascular AMD is termed geographic atrophy and is defined by substantial RPE loss and progressive degeneration of the proximal photoreceptors^31^. It is possible that early and progressive changes to RPE cells in AMD can be characterized by evaluating the microscopic spatial distribution of RPE fluorophores and/or changes in their AF spectra.

Adaptive optics ophthalmoscopy allows for cellular level imaging of the living retina^33,34^. Most often, it utilizes a closed-loop wavefront sensing (WFS) control that minimizes the wavefront aberrations to produce near diffraction-limited images of the retina. Direct imaging of the RPE has proven difficult, as the reflectance signal from the photoreceptors dominates the signal. Only optical coherence tomography (OCT) consistently visualizes all the retinal layers in cross-section but despite its impressive axial resolution (5–10 μm), individual cells cannot be distinguished in the RPE layer without AO (due to 10–20 μm lateral resolution) and substantial volumetric averaging^35,36^.

Fluorescence AOSLO has been used to image SWAF and IRAF in healthy volunteers, revealing the fine structure of the RPE cell mosaic *in vivo*^7,8^. However, reports are sparse, with only one other group showing images of the RPE obtained using IRAF to date^28^. Though they made morphometric measurements using their images in several eyes, they show only two images from normal eyes and one from a patient with retinitis pigmentosa. Most of the AO-IRAF using AOSLO has been done with 795 nm excitation with emission collected between 800–850 nm, as is done traditionally done with indocyanine green (ICG) imaging. Grieve *et al*. demonstrated AO-IRAF imaging with 757 nm excitation in several healthy eyes and although fine scale granularity of AF was seen in the sole AMD patient they imaged, individual cells were not^27^.

Here we demonstrate AO-IRAF imaging for RPE cell visualization in healthy eyes and eyes with AMD. We used a broadband super-luminescent diode (SLD) centered at 795 nm for excitation and observed emission in the band from 814 to 851 nm.

## Methods

### Participants

For this observational study, 22 participants were imaged: 7 young healthy volunteers and 15 patients with AMD. To be included in the study, AMD patients had to be over 55 years of age and have received a diagnosis of non-exudative AMD in at least one eye. The healthy volunteers were recruited through the research registry of the Clinical and Translational Science Institute of the University of Pittsburgh. Written informed consent was obtained from all participants following an explanation of experimental procedures and risks both verbally and in writing. Participants were compensated for their participation time. All experiments were approved by the University of Pittsburgh Institutional Review Board and adhered to the tenets of the Declaration of Helsinki. To ensure that imaging was safe, all light levels were kept below the limits imposed by the latest ANSI standard for safe use of lasers^25^.

### Optical design of the AOSLO

**Figure 1:**
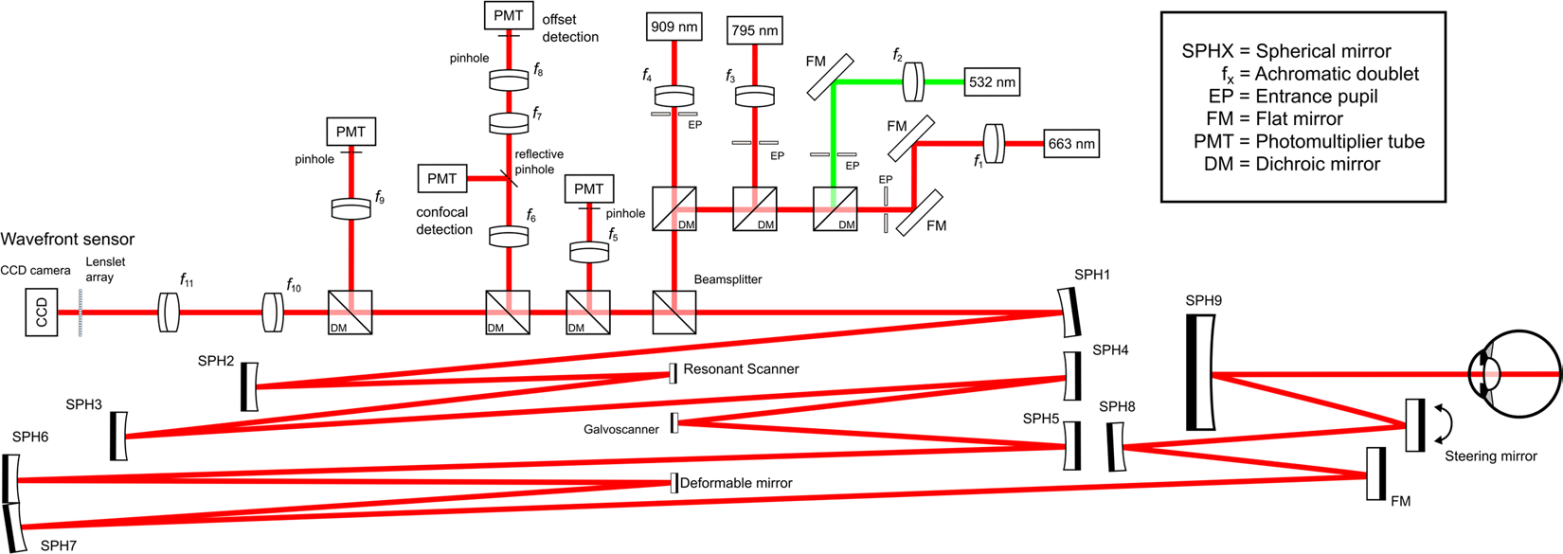
Schematical representation of the optical layout of the Pittsburgh AOSLO. All the achromatic doublets $({f}_{\text{x}})$ have focal length of 100 mm excluding the WFS arm where the beam is magnified by 0.75 to avoid beam clipping at the sensor. Before the beamsplitter, all the illumination channels are made co-axial with tip/tilt mounted mirror pairs. The first telescope magnifies the beam 0.5× and the second telescope magnifies the beam by a factor of 2. The remaining mirror telescopes maintain the entrance pupil diameter of 7.2 mm. On the detection side, each dichroic mirror peels off the desired wavelength band for detection which can be then further narrowed with a bandpass filter. For 795 mm confocal reflectance imaging the light is physically separated with a reflective pinhole in order to image single-scattered light (confocal) and multiply scattered light (offset detection) simultaneously. System here is not drawn to scale due to the optical design and beam path existing in three dimensions and keeping the components easily resolvable and distinct.

The AOSLO system built in Pittsburgh follows closely after a recent Rochester AOSLO optical design^37^. The major difference in our system is the lack of a widefield SLO and additional tip/tilt mirrors in the optical path for real-time optical stabilization. The optical relay path follows closely the afocal off-axis design principles of Dubra and Sulai^38^. The system has four sources for illumination and four detection channels with the current data collection configuration limited to a three channel simultaneous recording. In the following sections each aspect of the system is discussed in detail. The schematic of the system can be seen in Fig. 1. A pupil camera was used to easily align the participant in the chinrest as well as to monitor the lateral beam alignment during the measurement. The field of view (FOV) could be varied from 0.5 to 1.5 degrees and the data presented here were obtained with a 1.5° FOV. A projector was used to provide a fixation target. The target stimulus was generated and controlled using custom software, using elements of the Psychophysics toolbox^39^. The time-averaged optical power at the cornea for the light sources were 200 μW (795 nm), and 23 μW (909 nm).

#### Light Delivery

Light delivery consists of four different sources that are collimated using 100 mm focal length achromatic doublets (AC254-100-B, Thorlabs GmbH, Germany) and made co-axial using a pair of dichroic mirrors on 3-axis kinematic stages on each channel before reaching the 90/10 beamsplitter (10B20NC.2, Newport, Andover MA, USA). The collimated 532 source (FiberTec II™, Blue Sky Research, Milpitas CA, USA) can be used for SWAF excitation. SLDs centered at 663 nm (FWHM = 7 nm) (MS-670-GI6, Superlum, Dublin, Ireland) and 795 nm (FWHM = 15 nm) (MS-795-GI15, Superlum, Dublin, Ireland) can be used for reflectance imaging and/or AF excitation. To prevent any light leakage to the AO-IRAF detection channel, a bandpass filter was used to cut off the long wavelength tail of the 795 nm source (ET775/50x, Chroma, Bellows Falls VT, USA). Lastly, a 909 nm laser diode (QFLD-905-200S, Qphotonics, Ann Arbor MI, USA) was used as the WFS beacon. Collimating lenses were mounted on motorized translation stages that allowed for precise focusing of the imaging and AF excitation sources relative to the collimated WFS beacon to compensate for the longitudinal chromatic aberration (LCA) of the eye. The beam size was controlled with an aperture and for each channel it was set to 7.2 mm, corresponding to the size of the limiting aperture in the system (deformable mirror).

#### Beam relay path

After the beamsplitter, the first pair of spherical mirrors magnifies the beam by a factor of 0.5 and relays the pupil plane to the resonant scanner (SC-30, EOPC, Ridgewood NY, USA) operating at ~16 kHz. From there, the second pair of spherical mirrors magnifies the beam by a factor of two and relays the pupil plane to the slow scanner scanning at ~30 Hz (6210H, Cambridge Technology, Bedford MA, USA). The third telescope has no magnification while relaying the pupil plane to the deformable mirror (DM97-08, ALPAO, Montbonnot-Saint-Martin, France). After the deformable mirror the beam (pupil plane) is relayed to the steering mirror (SM) and from there it enters the eye after reflecting from a large 8″ diameter mirror (SPH9 in Fig. 1).

#### Light detection

The light emitted from the retina, whether it is scattered light or fluorescence, follows the same path back as illumination and is de-scanned before reaching the beamsplitter where 90% passes through in transmission. Dichroic mirrors are placed in the beam path to peel off different wavelength bands towards the different channels as seen in Fig. 1. The first dichroic mirror reflects everything below 757 nm (FF757-Di01, Semrock, Lake Forest IL, USA) and the beam is focused to the photo-multiplier tube (PMT; H7422-40, Hamamatsu Photonics, Japan) with an achromatic doublet (AC254-100-B, Thorlabs GmbH, Germany). For 663 nm confocal imaging, a double-stacked, air-gapped filter was used (FF01-661/20, Semrock, USA). The second dichroic mirror reflects everything below 810 nm (T810lpxr, Chroma, USA). The reflected beam is then focused with an achromatic doublet (AC254-100-B, Thorlabs GmbH, Germany) to a custom-made reflective pinhole (Opto-Line International Inc, Wilmington MA, USA). The central portion of the Gaussian beam is then directed to a second PMT (H7422-50) creating a confocal channel for the 795 nm beam. The remainder of the beam (outer portion) is relayed with an achromatic doublet pair (MAP10100100-B, Thorlabs GmbH, Germany) to a third PMT, creating a second retinal conjugate focal plane for offset imaging. In the main detection beam path, the third dichroic reflects everything below 875 nm (FF875-Di01, Semrock, USA) towards the fourth and final PMT that serves as the AO-IRAF detection channel. To make sure that only the autofluorescence signal is detected, a double-stacked ICG emission filter was used (FF01-832/37, Semrock, USA). Finally, the 909 nm light is magnified 0.5 times with a lens-based telescope before reaching the wavefront sensor. The Shack-Hartmann WFS was built using a CCD camera (GS3-U3-15S5M-C, FLIR, Wilsonville, OR, USA) and a microlens array with 203 μm lenslet pitch and a focal length of 6.7 mm (MLA-S203-f7, RPC Photonics, Rochester, NY, USA), giving a sampling value of 26 lenslets across the pupil at the sensor.

### Data acquisition

Custom electronics allowed the system to acquire data from three channels simultaneously. Based on the imaging protocol, appropriate channels could be selected. The field-programmable gate array (FPGA) card (Virtex ML506, Xilinx, San Jose CA, USA) was programmed to function as a specialized frame grabber, similar to Yang *et al*.^40^. The signal from the PMT was amplified and digitized to provide 8-bit grayscale images. The visible, NIR confocal and offset channels used the same amplifiers (C6438-01, Hamamatsu Photonics, Japan), while the AO-IRAF detection channel used one with slightly higher gain that was adjustable (TIA60, Thorlabs GmbH, Germany).

To optimize the confocality (maximizing mean pixel value in the image) and image quality in our system, the PMTs were placed on motorized xyz-stages allowing precise control of the pinhole location^7^. After qualitatively assessing the image quality and monitoring the mean pixel value, the Nelder-Mead simplex algorithm^41^ was used to find the optimum pinhole location computationally, as described previously^7^. This was especially important in the AO-IRAF detection channel as the signal is very weak and noisy, so optimizing the signal with the naked eye is impossible.

The data acquired in this study only used two of the four available light sources, namely 795 nm (for excitation) and 909 nm (for wavefront sensing). From the possible light detection channels, only the confocal 795 nm and NIR autofluorescence were used.

### Clinical imaging

All participants were imaged with other imaging modalities that are well-established. All the images (including AO-IRAF) were taken during the same visit if the time allowed. Otherwise two visits were scheduled that usually occurred within the same week. All images were obtained by the same ophthalmic photographer (one of the authors) to get consistent image quality for every participant. The clinical SLO/OCT imaging (Spectralis HRA + OCT, Heidelberg Engineering GmbH, Heidelberg, Germany) produced infrared autofluorescence (IRAF), blue light autofluorecence (BAF) and OCT images as well as infrared (IR) reflectance SLO images. Axial length was measured (IOLmaster, Carl Zeiss Meditec, Dublin CA, USA) to scale the images correctly across modalities. Flood-illumination AO fundus images were also obtained (rtx1-e, Imagine Eyes, Orsay, France).

### Image processing

AOSLO data were acquired at 30 Hz with 60–90 second exposures. The sinusoidal artifact in the images was corrected using previously published methods^42^. The confocal 795 nm channel was used to co-register the AF images. Images were registered using the strip-based image registration algorithm of Yang *et al*.^43^ or a similar strip-based registration algorithm developed in-house. The eye motion data from the confocal channel were used to co-register the data from the AO-IRAF channel as their data acquisition was synchronized^44^. The transverse chromatic aberration (TCA) was assumed to be negligible between 795 nm and the 814–851 nm emission range. However, it should be noted that TCA can still affect the image quality when the pupil moves, as has been shown for SWAF^9^.

Averaged images were montaged together manually using Adobe Photoshop CC (Photoshop CC, Adobe Systems Inc., USA). AO-IRAF images were linked with the confocal images to ensure accurate co-registration of each image modality. After montaging, the contrast and the brightness were adjusted for individual images to obtain a uniform contrast/brightness across the montage. After this, it was possible to add different modalities as separate layers in Photoshop to have an accurate co-registration of the images from different modalities. The foveal center was determined by using the OCT data to determine the precise location of the foveal pit and its corresponding position on the co-registered SLO and multi-modal images.

## Results

From the healthy participants, an expected mosaic of individual RPE cells was seen in 4 of 7 cases, with some showing a stronger mosaic pattern at the fovea than others. The rest did not show a distinct RPE mosaic pattern but rather observable AO-IRAF microstructure. The participants that showed more distinct patterns were in their early 20s, whereas older volunteers either did not show the expected cell mosaic pattern at all or showed a less distinct mosaic. One control was imaged at two different time points over the course of a week, displaying very similar RPE microstructure at both visits. Corresponding confocal images were high contrast with clearly resolved photoreceptors, suggesting that imperfect optical quality was not a factor limiting the visibility of the mosaic.

For the AMD patients, the typical RPE mosaic pattern was not observed in the same way as with the healthy controls and individual RPE cells were only clearly visible in some areas in four subjects. However, the AF emission patterns were markedly different from controls, showing AF clusters and large areas on the order of the size of several cells with hyper- or hypo-AF signal.

### Healthy volunteers

**Figure 2:**
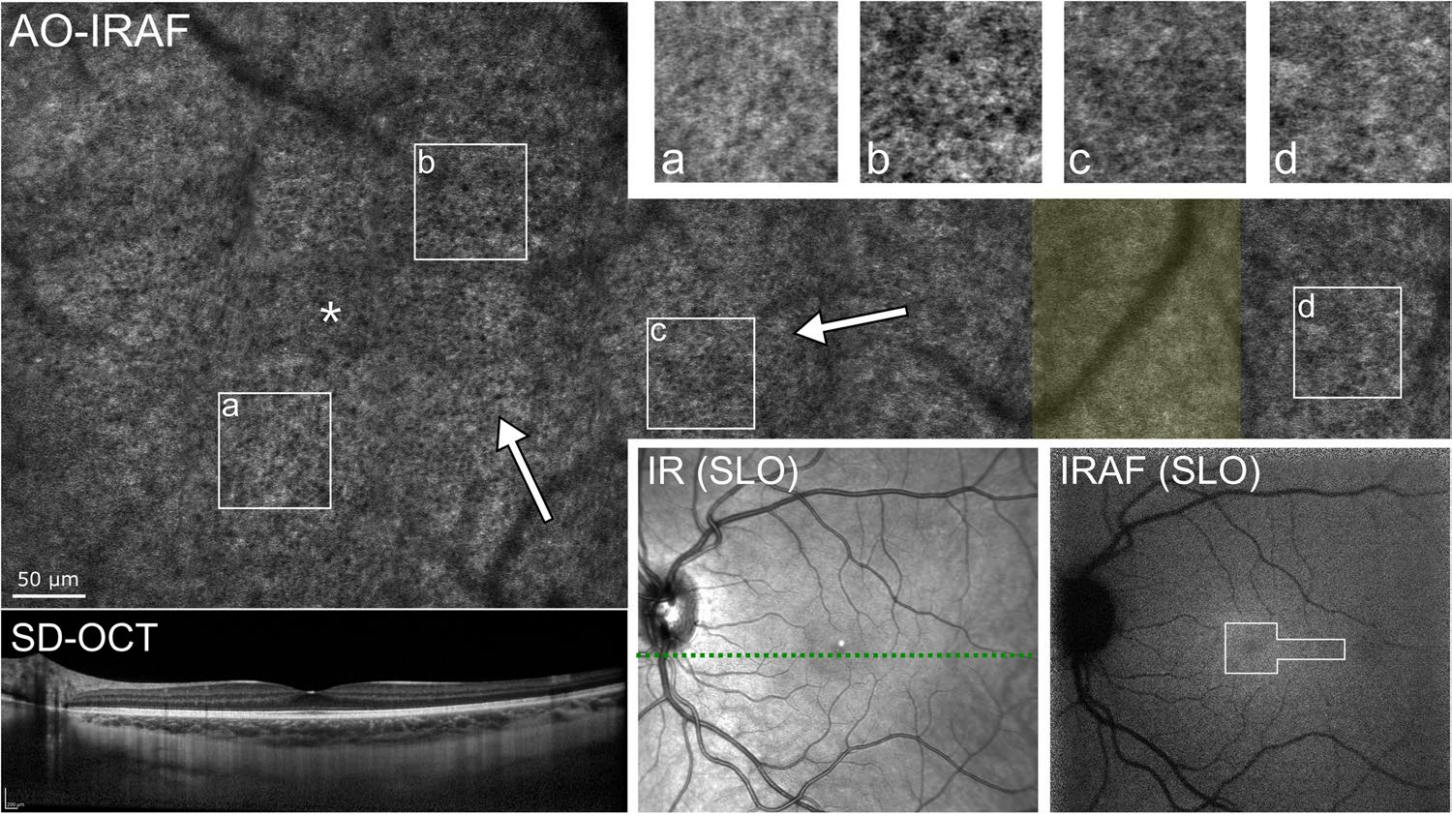
AO-IRAF images from a healthy volunteer (upper panels) compared with clinical images obtained with the Spectralis (lower panels). The IRAF SLO image shows a noisy but relatively uniform signal from the area of the fundus images with AOSLO. OCT shows no abnormalities. The fovea is marked in the AO-IRAF montage (above left) with an asterisk and the arrows indicate areas that show the expected hexagonal pattern of the RPE mosaic. The area marked with a transparent yellow square indicates a location where the imaging quality was sub-optimal. This resulted in poor image stabilization and reduced image quality in the averaged AO-IRAF image.

The RPE cell mosaic appearance in AO-IRAF varied substantially in the healthy volunteer group. Figure 2 shows an example of one participant that showed a well resolved mosaic of individual RPE cells that is observed in the fovea and remains visible to about 5° eccentricity (temporal). Some areas do not show the mosaic as clearly as others (Fig. 2).

**Figure 3:**
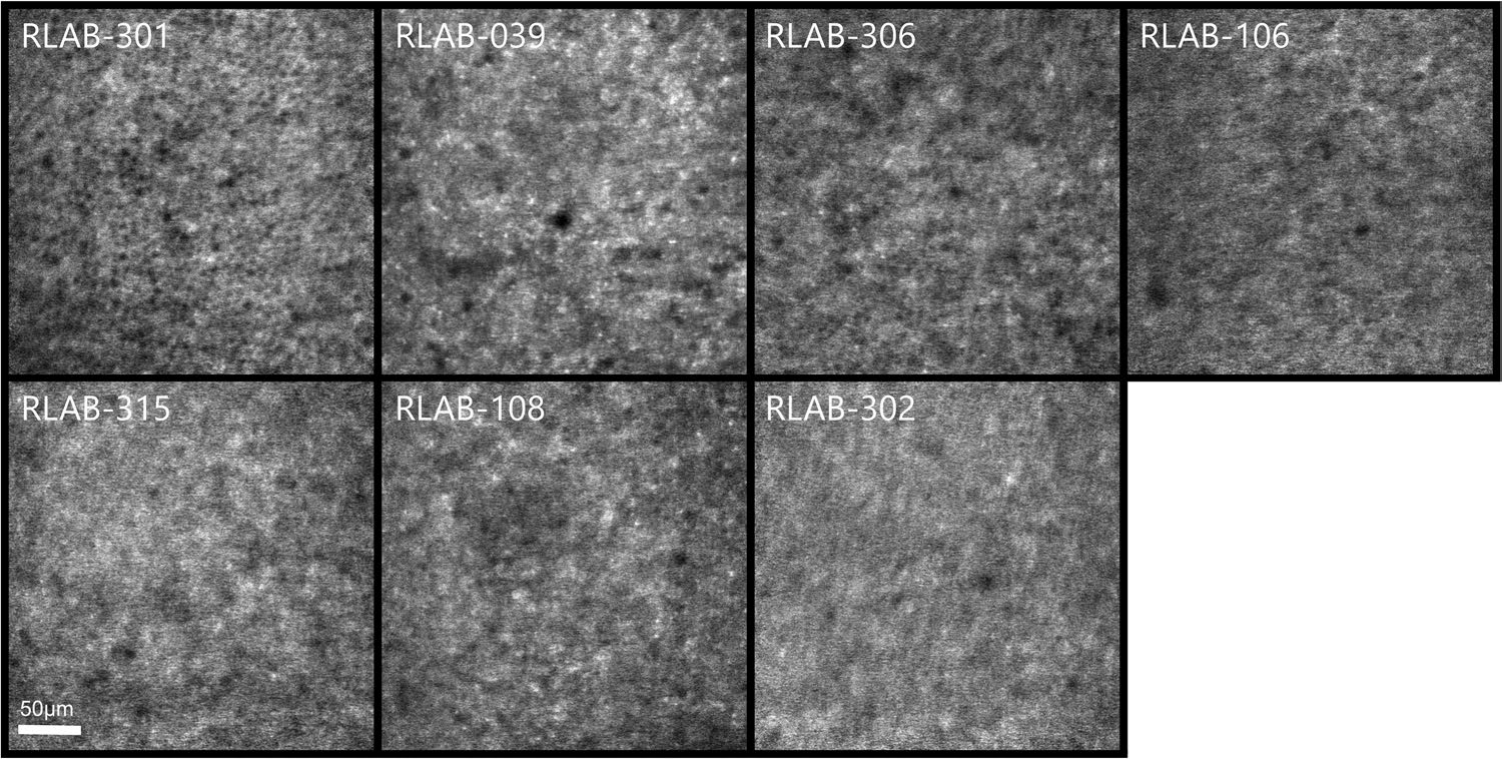
AO-IRAF signal variability in the fovea between healthy volunteers with normal vision. When comparing these images, there is a lot of variation in the signal. Some of the participants (e.g. 301) show a relatively clear cell mosaic, whereas others (e.g. 302 and 315) show pattered microstructures but lack the expected RPE cell mosaic. A strong AO-IRAF signal is seen with participant 039 but it does not manifest as the expected RPE mosaic but rather shows an intriguing microstructure with several hyper-reflective spots, on the order of the size of cones, that corresponded to positions where cones were clearly visible in the confocal photoreceptor image.

**Figure 4:**
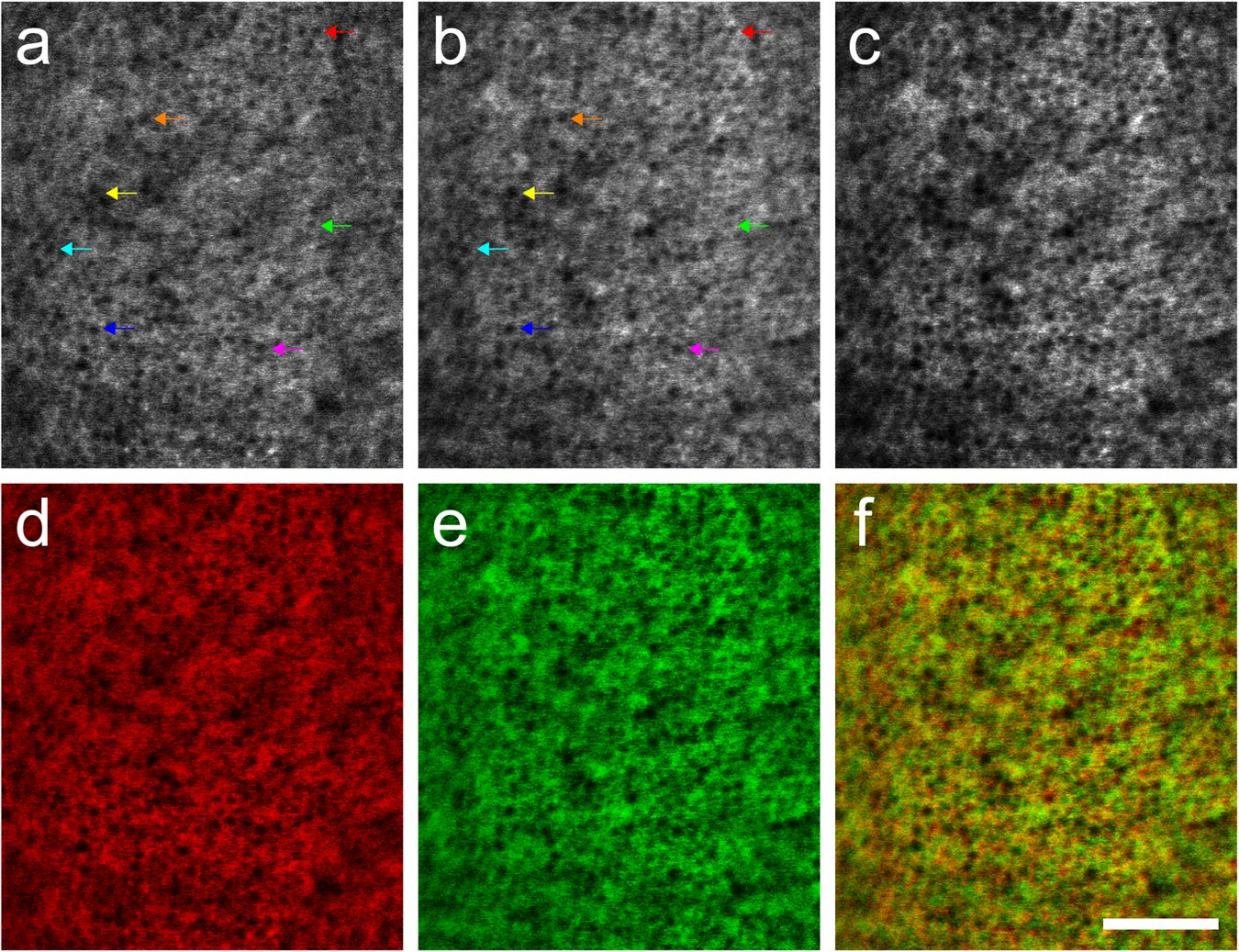
AO-IRAF images taken from fovea of the same healthy eye (RLAB-301). The same individual RPE cells were visible in AO-IRAF, despite some variability in the AF signal strength across each image (**a**,**b**). Colored arrows point out a few of the individual RPE cells that were visible in both of these two images taken one week apart. A multiplication of the images from the two time points (**c**) demonstrates correspondence between the images and shows an improved SNR and cell visibility across the field of view. False colored versions of (**a** and **b**) are shown in (**c** and **d**), respectively and are color-merged in (**f**) to appreciate small differences between the cells when imaged at the two time points. Scale bar 100 μm.

Figure 3 shows the variability of the foveal AO-IRAF signal among the healthy volunteers with normal vision. Some of the participants show expected RPE mosaic whereas some of the participants show patterns of what appear to be well-resolved AF microstructure but not a clear pattern tracing out the boundaries of individual RPE cells. Interestingly, the corresponding confocal reflectance image for each participant from the exact same area showed a well resolved photoreceptor mosaic, suggesting good AO performance and image quality. Figure 4 shows the repeatability of the AO-IRAF signal on a healthy volunteer. Two timepoints are shown over a period of one week, showing similar structure and signal level.

### Patients with AMD

**Figure 5:**
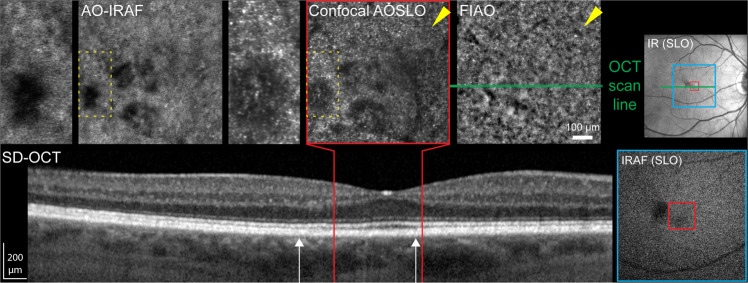
Age-related macular degeneration in a 65 y/o male imaged with AO-IRAF (RLAB-102). On SD-OCT, the outer hyperreflective band corresponding with the interdigitation zone is discernible outside the foveola, but cannot be identified within the region bounded by the white arrows. IRAF in SLO (lower right) shows mild attenuation of the IRAF signal in the region where we see marked loss in well circumscribed regions in the AO-IRAF image. The confocal AOSLO image shows significant alterations in the photoreceptor cell structure in areas where the AO-IRAF signal is attenuated in AOSLO (dashed yellow rectangle). In some areas cones were less visible in the confocal AOSLO image than they were in the flood-illumination adaptive optics image, perhaps due to cones being misaligned or having altered outer segments (yellow arrowheads).

Autofluorescence microstructure was visible in most AMD patients but individual RPE cells were often not well resolved. In many cases, AO-IRAF signal exhibited pronounced heterogeneity. Near the fovea, there was loss of the AO-IRAF signal across regions spanning tens to hundreds of microns. The corresponding OCT scan showed that the RPE appeared intact in this area but that the interdigitation zone was indistinct from the RPE band (Fig. 5). The confocal AOSLO image showed alterations in retinal structure that were clearly visible at the photoreceptor layer and these manifest as areas of reduced reflectance (e.g. Fig. 5, dashed yellow rectangle).

Corresponding alterations in these areas are even more pronounced in the AO-IRAF image, where the AO-IRAF signal is absent in some of the areas. These manifest as areas with reduced signal in the noisy conventional IRAF (Fig. 5, red square). AOSLO images revealed the photoreceptor layer irregularities more clearly than flood-illuminated adaptive optics (FlAO) imaging across most of the image. The reduced lateral and axial resolution (4–5 μm lateral and approx. 52 μm focus depth for 5 mm pupil) of the flood illumination system may account for these differences. However, in some regions where the cones were not clearly visible in the confocal image (Fig. 5, arrows), they were visible in the flood-illumination image; this is possibly due to the increased depth of focus of the flood-illumination system being capable of detecting cones when they are misaligned or when their outer segments are altered.

**Figure 6:**
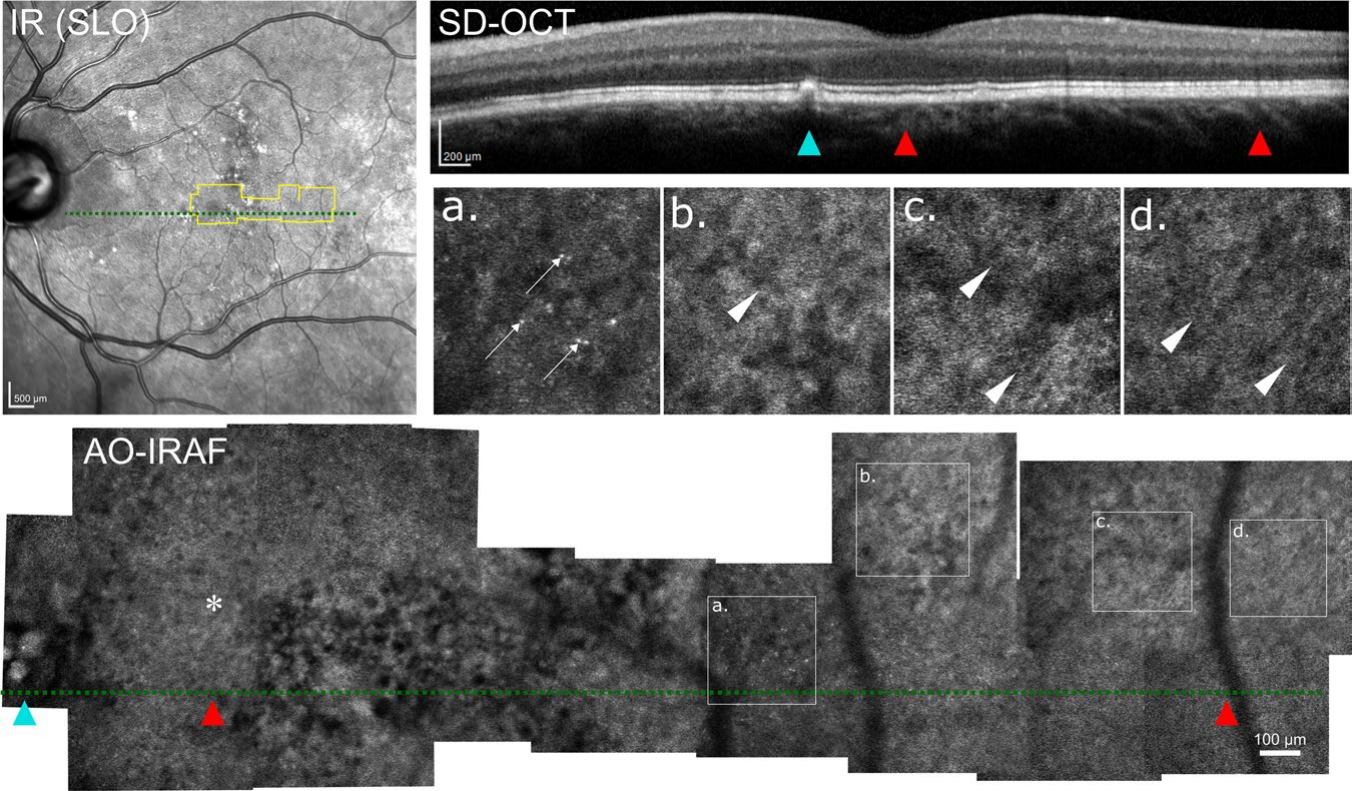
A 74 y/o AMD patient imaged with AO-IRAF (RLAB-189). No distinct mosaic of RPE cells was seen near the fovea (center marked with asterisk), but rather a pattern of distinct hyper- and hypo-AF was seen, suggesting substantial alterations to the RPE fluorophores. The area corresponding to the AO-IRAF montage is outlined in the SLO reflectance image with yellow borders and the location of the B-scan is marked with the green dashed line. Red arrowheads denote corresponding points in the SD-OCT B-scan and AO-IRAF montage. The small drusenoid deposit seen temporal to the fovea on OCT corresponds with the microstructure seen in the AO-IRAF montage (marked with cyan arrowhead). The interdigitation zone is indistinct on OCT in the area of the fovea where the pattern of large areas of hyper- and hypo-AF are most pronounced. In some locations, fluorescent structures were seen that co-localized with cones in the reflectance image (some are marked with white arrows). In some peripheral locations a faint mosaic of individual RPE cells could be seen (white arrowheads). (**a**–**d**) Zoomed in regions of interest from the montage.

Another AMD patient is shown in Fig. 6. The AO-IRAF montage shows a faint RPE mosaic in the more temporal areas (Fig. 6, arrowheads). The area in and around the fovea shows a pattern of microstructure with large areas of hypo and hyper-AF that are on the order of the size of many RPE cells, suggesting redistribution and/or loss of the AO-IRAF fluorophores. In the OCT B-scan the interdigitation zone band is indistinct but the RPE band appears intact in this areas. The AO-IRAF montage in Fig. 6 also shows a distinctive cone signature in some locations (few marked with white arrows). There are several bright spots seen in the image and when we compared them to the overlaid confocal AOSLO, they were co-localized with cones.

It is worth noting that in both of these AMD examples, we saw loss of AO-IRAF signal in the fovea that corresponded to areas where the RPE band appeared intact but the interdigitation zone was indistinct on OCT. The single outer retinal band that we see in these locations appears thickened in this area and appears roughly the same thickness as the two outer bands combined where they appear distinct at more peripheral locations. Moreover, any volume loss, which might indicate complete “loss” of the interdigitation zone, is not appreciated in this area.

**Figure 7:**
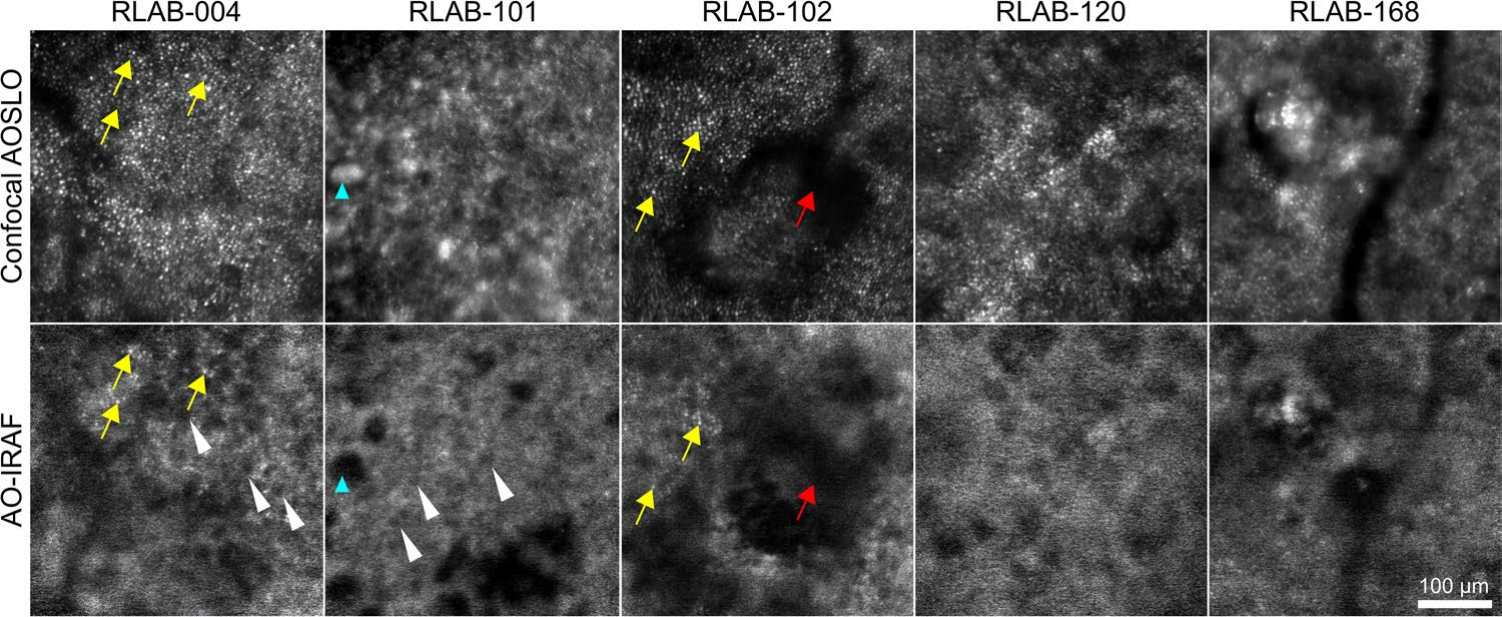
Five different patients showing a patterns of AO-IRAF microstructure suggesting a redistribution and/or loss of RPE fluorophores. The white arrowheads denote a few locations where individual RPE cells are visible (RLAB-004 and RLAB-101). The yellow arrows mark the areas where the cone signature is seen in the AO-IRAF images with the corresponding area marked in the confocal AOSLO images. Cyan arrowhead points to an example area with highly reflective structure in the confocal image with AO-IRAF image showing absence of AF signal. In RLAB-102 the red arrow indicates an example area with no signal in either channel, potentially showing altered cone mosaic in confocal channel correlating with altered AF signal. Neither individual RPE cells nor cone signatures were seen in the AO-IRAF images of RLAB-0120 and RLAB-0168, despite well resolved cones in the confocal image and fine microstructural details visible in the AO-IRAF images.

Figure 7 shows confocal AOSLO and AO-IRAF images from five different AMD patients. Despite areas of clearly visible cones in all the corresponding confocal images, RPE cell visibility varied markedly with individual RPE cells (Fig. 7, arrowheads) only seen in some eyes. Hyper-AF cone signatures were often observed in the AO-IRAF images where cones were visible at corresponding points in the confocal image (Fig. 7, yellow arrows). The patterns observed in the AO-IRAF signal are suggestive of either redistribution of fluorophores and/or loss of these organelles (e.g. location marked with blue arrowhead in Fig. 7). Areas where there is no AF or low AF may also be areas where the cone alterations seen in confocal AOSLO in corresponding areas may be altering the AF (e.g. location marked with red arrow in Fig. 7).

## Discussion

AOSLO remains challenging in patients with AMD as the optical quality of the eye naturally degrades over time and this can seriously impact image quality in adaptive optics ophthalmoscopy in older eyes. Cataracts can challenge the stability of the adaptive optics correction and this can be exacerbated when imaging older eyes that often have smaller pupils even after pharmacological mydriasis. Further, the structural alterations seen in patients whose retina is severely altered by AMD, and increased eye motion due to poorer vision, can reduce the performance of image registration resulting in fewer images of lower quality available for averaging. However, despite these difficulties, these results provide tantalizing glimpses into how RPE AO-IRAF fluorophores are distributed in AMD. Further optimization of parameters such as the excitation wavelength and the emission band may yield improved results with severely diseased eyes yet it is possible that the redistribution of fluorophores both with aging and in AMD may make routine visualization of the RPE cell mosaic challenging no matter what AF bands are used.

Though most AMD patients did not exhibit the expected RPE mosaic with AO-IRAF that we have seen previously in some healthy eyes using both AO-IRAF and SWAF and in our previous study of a small number of AMD eyes with SWAF^7,9^, the montages show interesting microstructural differences compared to normal young healthy eyes. It appears as though AO-IRAF fluorophores can be redistributed in AMD patients, particularly in the foveal region. Fluorophore compartmentalization within the RPE may be altered in AMD, making it difficult to see the same type of structure of the RPE cells in the AO-IRAF images compared to healthy volunteers. Additionally, substantial fluorophore redistribution may exist before alterations are visible in other imaging modalities suggesting AO-IRAF may provide insight to early RPE changes in AMD. Moreover, it is still unclear what changes in the RPE in older eyes is due to natural variation in the AO-IRAF signal and what is related to AMD. To address this, a larger patient cohort is needed with age-matched controls to fully characterize the differences. Finally, multimodal imaging, including OCT, is essential when interpreting the AO-IRAF images. The damaged RPE layer manifests as increased light penetration to the choroid and these areas can be then assessed by observing the AO-IRAF images. The OCT B-scans that were taken from the affected area showed very minimal disruptions in the RPE whereas the AO-IRAF signal showed significant alterations. Interestingly, in the foveal areas where we saw larger areas of hypo- and hyper-AF, the interdigitation zone appeared indistinct on OCT. In these areas, cone signal was reduced in confocal AOSLO, suggesting that the cone outer segments and/or their interdigitation with the apical processes of the RPE cells was altered in these areas. Because we have shown previously that cones appear to sometimes modulate the AO-IRAF signal, it is possible that some alterations in the pattern of the AO-IRAF signal may be driven by alterations in the cones, though this hypothesis needs further study.

Despite having double-stacked bandpass filters before the PMT as well as in front of the excitation beam to eliminate any reflectance leak in the AO-IRAF channel, a cone signature was visible in AO-IRAF channel for some healthy controls and AMD patients. As we went to great lengths to prevent any light leakage from the excitation beam, we do not think this is from reflectance light leaking into the IRAF channel. The most plausible explanation for these fluorescent cone signatures is the hypothesis that cones^45,46^ are modulating the AO-IRAF signal. However, it is not fully clear if this is due to the waveguide properties of the cones or if it is driven by a modulation of the excitation light going in or for the fluorescence emitting from the RPE. It is also possible that absorption of light by the cone photopigments plays some role in this phenomenon. An alternative hypothesis is that the cones themselves are fluorescent in the NIR. However, there is no evidence for a candidate fluorophore residing in the cones that would be excitable with NIR light. For the areas where the AO-IRAF signal is very low, and the corresponding confocal image shows altered cone reflectance, it is possible that the photoreceptor alterations are contributing to the loss of the signal. Therefore, some of the alterations seen in the pattern of AO-IRAF, particularly in AMD eyes, may be due to alterations in the properties of the overlying cones. Further studies are needed to fully characterize the extent of these fluorescent cone-like structures and see if there is a correlation e.g. with age or location and if there are special circumstances where the cones are more often visible in the AO-IRAF image.

The 795 nm excitation light is used often in AO-IRAF imaging. Based on the *in vitro* experiments with synthetic melanin or melanin extracted from *Sepia*, the emission peak with 785 nm light is located in the 850–900 nm range^20^. Therefore, it is likely that a major portion of the signal is coming from the melanin. However, the recent work of Taubitz *et al*. suggests that in aged eyes, lipofuscin exhibits both SWAF and NIRAF. This is supported by our previous work showing co-localization of the fluorescence signal in healthy and relatively young eyes^9^. How this co-localization varies with age remains to be explored as the AO-IRAF signal and spectra might vary significantly between younger and older eyes. It is likely that natural age-related alterations in the relative abundance and composition of RPE AF organelles impacts the efficacy of different AF wavelength bands for visualizing RPE cells across the lifespan.

## Conclusions

This observational study demonstrates the potential of AO-IRAF imaging applied to patients with AMD. Although a clear RPE mosaic was often not revealed, even in normal eyes, AO-IRAF in AMD showed marked and substantial microstructural differences compared to normal eyes. Since it is possible that the AF signal is not only originating from melanin, but also lipofuscin, future study should evaluate changes in RPE AF spectra with aging. Considering the low level of AF coming back to the detector, doubling or even tripling the excitation power is warranted as this is allowed by the laser safety standard due to the large safety margin provided by NIR light. Finally, these results warrant validation in a larger patient cohort and in normal individuals across the lifespan to establish normal variability and to distinguish normal age-related changes in AO-IRAF from those related to disease.
